# Analyses of ‘change scores’ do not estimate causal effects in observational data

**DOI:** 10.1093/ije/dyab050

**Published:** 2021-06-07

**Authors:** Peter W G Tennant, Kellyn F Arnold, George T H Ellison, Mark S Gilthorpe

**Affiliations:** Leeds Institute for Data Analytics, University of Leeds, Leeds, LS2 9NL, UK; Faculty of Medicine and Health, University of Leeds, Leeds, LS2 9LU, UK; Alan Turing Institute, British Library, London, NW1 2DB, UK; Leeds Institute for Data Analytics, University of Leeds, Leeds, LS2 9NL, UK; Faculty of Environment, University of Leeds, Leeds, LS2 9JT, UK; Leeds Institute for Data Analytics, University of Leeds, Leeds, LS2 9NL, UK; Faculty of Medicine and Health, University of Leeds, Leeds, LS2 9LU, UK; Centre for Data Innovation, Faculty of Science and Technology, University of Central Lancashire, Preston, PR1 2HE, UK; Leeds Institute for Data Analytics, University of Leeds, Leeds, LS2 9NL, UK; Faculty of Medicine and Health, University of Leeds, Leeds, LS2 9LU, UK; Alan Turing Institute, British Library, London, NW1 2DB, UK

**Keywords:** Analysis of change, change scores, difference scores, gain scores, change-from-baseline variables, directed acyclic graphs

## Abstract

**Background:**

In longitudinal data, it is common to create ‘change scores’ by subtracting measurements taken at baseline from those taken at follow-up, and then to analyse the resulting ‘change’ as the outcome variable. In observational data, this approach can produce misleading causal-effect estimates. The present article uses directed acyclic graphs (DAGs) and simple simulations to provide an accessible explanation for why change scores do not estimate causal effects in observational data.

**Methods:**

Data were simulated to match three general scenarios in which the outcome variable at baseline was a (i) ‘competing exposure’ (i.e. a cause of the outcome that is neither caused by nor causes the exposure), (ii) confounder or (iii) mediator for the total causal effect of the exposure variable at baseline on the outcome variable at follow-up. Regression coefficients were compared between change-score analyses and the appropriate estimator(s) for the total and/or direct causal effect(s).

**Results:**

Change-score analyses do not provide meaningful causal-effect estimates unless the baseline outcome variable is a ‘competing exposure’ for the effect of the exposure on the outcome at follow-up. Where the baseline outcome is a confounder or mediator, change-score analyses evaluate obscure estimands, which may diverge substantially in magnitude and direction from the total and direct causal effects.

**Conclusion:**

Future observational studies that seek causal-effect estimates should avoid analysing change scores and adopt alternative analytical strategies.

Key Messages

- ‘Change scores’ provide a simple summary measure of the average change in a variable between two time points; they are commonly used when analysing ‘change’ in an outcome with respect to a baseline exposure.
- Analyses of outcome-change scores do not estimate causal effects except under randomized experimental conditions; in some (non-randomized) situations, the implied ‘effect’ may be of the opposite sign to the total and/or direct causal effect.
- Future observational studies that seek causal-effect estimates should avoid analysing outcome-change scores and adopt alternative analytical strategies; studies that have conducted analyses of outcome-change scores should be viewed with caution and their recommendations revisited.

## Introduction

Studies of change are a cornerstone of research in the health sciences. Understanding the natural history of disease, and in turn predicting prognoses, is of enormous interest to physicians and patients alike. Analyses of ‘change’ are, however, deceptively complex in observational data. One of the most common, yet poorly recognized, challenges stems from the use and interpretation of ‘change scores’.

Change scores (e.g. $\Delta Y=Y_{1}-Y_{0}$), also known as ‘difference scores’, ‘gain scores’ and ‘change-from-baseline variables’, are composite variables that have been constructed from repeated measures of a single parent variable (Y) by subtracting a subsequent measure of the parent ($Y_{1}$, ‘follow-up’) from a prior measure ($Y_{0}$, ‘baseline’). The resulting composite variable retains information from both of its determining parents and hence will share a tautological association with either if analysed by regression or correlation.^1^ This was first recognized by Oldham in 1962, who demonstrated that an association averaging $r=\pm 1/\sqrt{2}$ occurs between either of the parent variables (i.e. $Y_{0}$ or $Y_{1}$) and their difference (i.e. $Y_{1}-Y_{0}$) if both have similar variances but are otherwise unrelated.^2^ This phenomenon explains the ‘law of initial value’ as a consequence of the sign disagreement between the baseline parent variable ($Y_{0}$) and its transformation in the composite change score ($-Y_{0}$), and is distinct from regression-to-the-mean.^1^

Relatively few analyses of change scores, however, involve straightforward tautological associations. More often, change scores are used as outcome variables in relation to a separate baseline treatment or exposure $X_{0}$ (e.g. ‘How do beta-blockers affect change in blood pressure?’). One of the most widely recognized issues in this context is the discordance between change-score analyses (i.e. where the outcome-change score ΔY is regressed on the baseline exposure $X_{0}$) and analyses of covariance (ANCOVA; i.e. where the follow-up outcome $Y_{1}$ is regressed on the baseline exposure $X_{0}$ and ‘adjusted for’ the baseline outcome $Y_{0}$).^3^^,^^4^ For example, Senn (2006) and Van Breukelen (2006) found that change-score analyses and ANCOVA provide similar and unbiased estimates when the exposure is randomized but provide ‘contradictory results’ when the exposure is not randomized. Frederick Lord’s eponymous paradox centres on this same ‘contradiction’ and the lack of an obvious ‘correct’ answer.^5^

Although studies of change are extremely common, the concept of change—and the use of change scores as a putative measure thereof—has received relatively limited formal consideration within a causal framework. Causal diagrams such as directed acyclic graphs (DAGs) provide a useful framework for understanding some challenges associated with observational data analysis, but they have not often been used to consider analyses of change scores specifically. Of the exceptions, Glymour *et al.* (2006) focused on the role of measurement error, arguing that analyses of outcome-change scores provide unbiased causal-effect estimates in some cases, but that error can be introduced by conditioning on the baseline outcome.^6^ Conversely, Shahar and Shahar (2010) argue that change scores are ‘not of causal interest’ and that 'modelling the change between two time points is justified only in a few situations’.^7^

The present article aims to provide an accessible explanation of why analyses of change scores do not estimate causal effects in observational (i.e. non-randomized) data and illustrate the potentially misleading consequences of doing so.

### Change scores do not represent exogenous change

In this section, we consider the concept of ‘change’ using DAGs. We focus on ‘exogenous change’ in an outcome variable (Y), which represents the structural (i.e. non-random) component of the follow-up outcome ($Y_{1}$) that has not been determined at baseline ($Y_{0}$) and can therefore potentially still be modified after baseline.

DAGs are semi-parametric graphical representations of hypothesized causal relationships between variables.^8^ Variables or events (depicted as nodes) are connected by unidirectional arcs (depicted as arrows), representing the presence and direction—though neither the nature nor the magnitude—of each hypothesized causal relationship. A path is a collection of one or more arcs that connect two nodes and a causal path is one in which all constituent arcs flow in the same direction. No variable can cause itself. By convention, we depict deterministic variables as double-outlined nodes.^9^

**Figure 1.**
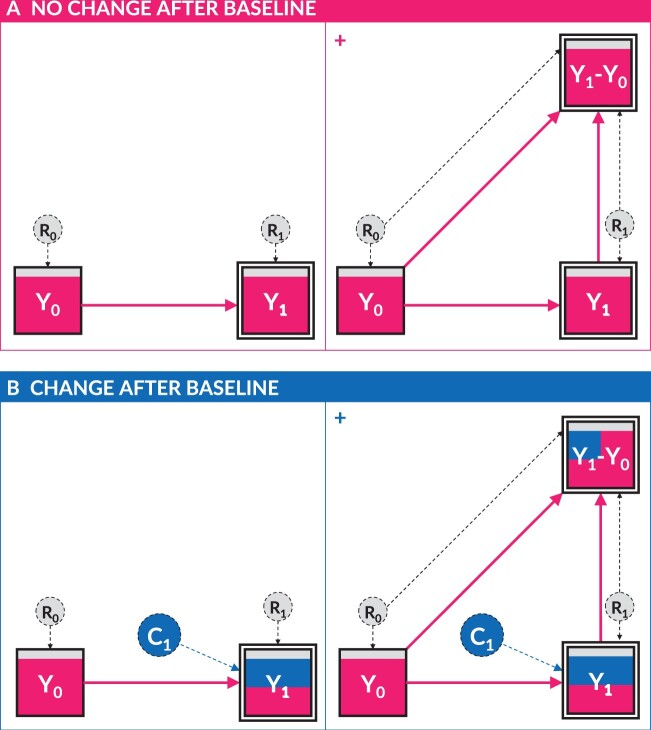
Directed acyclic graphs (DAGs) depicting the relationship between an outcome variable at baseline ($Y_{0}$) and follow-up ($Y_{1}$), where the follow-up measure is completely determined. In panel A, the values of $Y_{1}$ are fully determined by $Y_{0}$ (and random processes $R_{1}$), so there exists no exogenous change. In panel B, the values of $Y_{1}$ are partly determined by $Y_{0}$ (and random processes $R_{1}$) and partly determined by exogenous factors representing ‘change’ ($C_{1}$). $C_{1}$, $R_{0}$ and $R_{1}$ are depicted as dashed (latent) variables, as they cannot be directly measured and are encapsulated within their descendent variables $Y_{1}$, $Y_{0}$ and $Y_{1}$, respectively. Panels A+ and B+ depict the same causal scenarios as panels A and B, respectively, but also show the composite change-score variable ($Y_{1}-Y_{0}$), which itself is completely determined by $Y_{0}$ and $Y_{1}$.

We first consider the simple example of repeated measures of an outcome variable (Y) that only fluctuate due to randomness (R) (see Figure 1, panel A). Values of the follow-up ($Y_{1}$) are entirely determined by the baseline ($Y_{0}$) plus the random features at follow-up ($R_{1}$). In this scenario, $Y_{1}$ cannot be modified except by modifying $Y_{0}$; no exogenous change exists. This is obvious in repeated measures of a fixed variable, such as height in healthy middle-aged adults. Although each individual’s height values $Y_{0}$ and $Y_{1}$ would likely differ slightly due to the random features at baseline ($R_{0}$) and follow-up ($R_{1}$), this only dilutes the observed relationship between $Y_{0}$ and $Y_{1}$_._ In the population, there would be no *overall* change in the average values of height at baseline and follow-up, and this would be correctly reflected by a change score with a mean of zero (Figure 1, panel A+).

The same causal scenario (i.e. Figure 1, panel A) could also describe repeated measures of a *dynamic* variable, whereby follow-up values are *fully determined* by baseline values via an algebraic function. As an example, consider the total expected number of radioactive particles Y in a sample of (non-depleted) uranium rods at some future point in time ($Y_{1}$), which may be estimated without bias from the current observed number of radioactive particles ($Y_{0}$) by the Universal Law of Radioactive Decay.^10^ The total observed value of Y would irrefutably change between $Y_{0}$ and $Y_{1}$, and each individual uranium rod would have a negative change score (the magnitude of which would increase with the size of $Y_{0}$). Nevertheless, no exogenous change exists; as previously, $Y_{1}$ cannot be modified except by modifying $Y_{0}$.

Finally, we consider a more realistic dynamic variable (Y), whose future values ($Y_{1}$) are only partly determined by the past values ($Y_{0}$), with the remainder determined by random features ($R_{1}$) plus other exogenous change ($C_{1}$) (see Figure 1, panel B). Here, $C_{1}$ represents all *non-random* changes in Y that are not pre-determined by $Y_{0}$, and so the concept of exogenous change can thus be considered an average of all the processes in $C_{1}\to Y_{1}$. In reality, $C_{1}$ is an unmeasurable, ongoing latent process whose value is only defined once the point of follow-up is fixed (in the same way as ‘age’ is undefined until the date of measurement is defined). Thus, the exogenous change between two time points is fundamentally encapsulated within, and can only be determined from, $Y_{1}$.

We do not specify the time window between $Y_{0}$ and $Y_{1}$, but it seems plausible that change could also be introduced after baseline by altering the *effect* of $Y_{0}$ on $Y_{1}$. This is equivalent to creating an intermediate node ($Y_{0.5}$) along the path between $Y_{0}$ and $Y_{1}$ that provides a later chance to modify $Y_{1}$ without invoking exogenous change. However, this only serves to delay the distinction between the determined and change components of $Y_{1}$, since, after $Y_{0.5}$, there is again no means to alter $Y_{1}$ other than through exogenous change. In theory, we could introduce another node and another, but eventually we would reach the node immediately prior to $Y_{1}$ in time ($Y_{1-\delta t}$), at which point there is no way to intervene in the effect of $Y_{1-\delta t}$ after $Y_{1-\delta t}$, and exogenous change is the only way to introduce change in Y.

### Isolating exogenous change with respect to a baseline exposure

The causal effect of a baseline exposure $X_{0}$ on ‘change’ in Y hence corresponds to the effect of $X_{0}$ on ‘exogenous change’ in Y, i.e. the structural part of $Y_{1}$ that has not already been determined by $Y_{0}$. This quantity can be expressed as the effect of $X_{0}$ on $Y_{1}|Y_{0}$ or the estimand $\alpha_{1}=\left\{E\left[Y_{1}|do\left(X_{0}=x_{0}\right),Y_{0}=y_{0}\right]-\;E\left[Y_{1}|do\left(X_{0}={\overset{´}{x}}_{0}\right),\;Y_{0}=y_{0}\right]\right\}$, where $x_{0}$ and ${\overset{´}{x}}_{0}$ are two contrasting levels of the exposure. This effect may be estimated by constructing, e.g., a regression model of the form $\hat{Y_{1}}={\hat{\alpha }}_{0}+{\hat{\alpha }}_{1}X_{0}+{\hat{\alpha }}_{2}Y_{0}$, which we refer to as the **follow-up adjusted for baseline analysis**, where ${\hat{\alpha }}_{1}$ represents the estimate for the estimand of interest ($\alpha_{1}$).

Construction and analysis of a change score likely represent an attempt to isolate this same effect from the apparent ‘effect’ of $X_{0}$ on $\Delta Y=Y_{1}-Y_{0}$ or the estimand $\beta_{1}=\;\left\{E\left[Y_{1}-Y_{0}|do\left(X_{0}=x_{0}\right)\right]-\;E\left[Y_{1}-Y_{0}|do\left(X_{0}={\overset{´}{x}}_{0}\right)\right]\right\}$, where $x_{0}$ and ${\overset{´}{x}}_{0}$ are again two contrasting levels of the exposure. This quantity may be estimated by constructing a regression model of the form $\hat{\Delta Y}={\hat{\beta }}_{0}+{\hat{\beta }}_{1}X_{0}$, which we refer to as the **change-score analysis** and where ${\hat{\beta }}_{1}$ represents the coefficient that is often (mis)interpreted as estimating the true effect of interest ($\alpha_{1}$). Instead of ‘standardizing’ $Y_{1}$ relative to $Y_{0}$, the change-score approach treats two separate events (i.e. $Y_{0}$ and $Y_{1}$) as one, thereby conflating the causal pathways involved. This can be seen by rewriting the estimand in full as $\beta_{1}=\;\left\{E\left[Y_{1}|do\left(X_{0}=x_{0}\right)\right]-E\left[Y_{1}|do\left(X_{0}={\overset{´}{x}}_{0}\right)\right]-E\left[Y_{0}|do\left(X_{0}=x_{0}\right)\right]+\;E\left[Y_{0}|do\left(X_{0}={\overset{´}{x}}_{0}\right)\right]\right\}$, which depends jointly on elements of the effects of $X_{0}$ on both $Y_{0}$ and $Y_{1}$, including the negative of the total causal effect of $X_{0}$ on $Y_{0}$.

**Figure 2.**
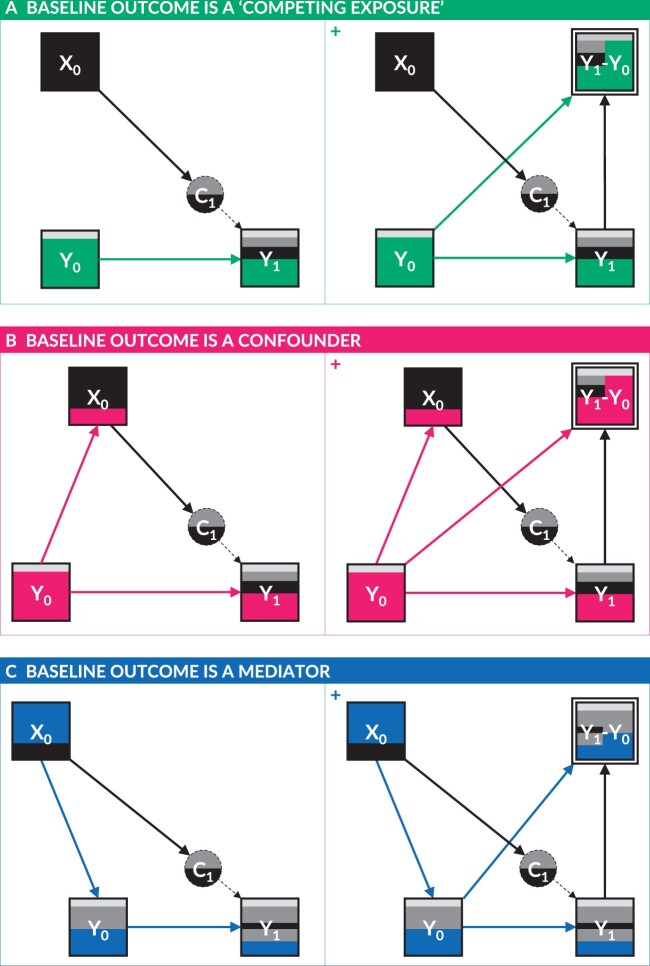
Directed acyclic graphs (DAGs) depicting three causal scenarios for analyses of change in an outcome (Y) in relation to a baseline exposure ($X_{0}$). Panel A represents a scenario in which the baseline outcome ($Y_{0}$) is a ‘competing exposure’ for the total causal effect of $X_{0}$ on the follow-up outcome ($Y_{1}$), i.e. $X_{0}$ is unrelated to $Y_{0}$ as in a well-conducted randomized experimental study. In this scenario, the total causal effect of $X_{0}$ on $Y_{1}$ is identical to the total causal effect of $X_{0}$ on ‘exogenous change’ in the outcome ($C_{1}$). Panel B represents a scenario in which $Y_{0}$ is a confounder for the total causal effect of $X_{0}$ on $Y_{1}$. In this scenario, the total causal effect of $X_{0}$ on $Y_{1}$ is again identical to the total causal effect of $X_{0}$ on $C_{1}$. Panel C represents a scenario in which $Y_{0}$ is a mediator for the total causal effect of $X_{0}$ on $Y_{1}$. In this scenario, the direct causal effect of $X_{0}$ on $Y_{1}$ that is not mediated through $Y_{0}$ is identical to the total causal effect of $X_{0}$ on $C_{1}$. Panels A+, B+ and C+ depict the same causal scenarios as panels A, B and C, respectively, but also depict the composite change score variables ($Y_{1}-Y_{0}$), which are completely determined by $Y_{0}$ and $Y_{1}.$

The degree of discordance between these two estimands ($\alpha_{1}\;$and $\beta_{1}$), and hence the coefficients in the *follow-up adjusted for baseline analysis* (${\hat{\alpha }}_{1}$) and the *change-score analysis* (${\hat{\beta }}_{1}$), will depend on the strength of the association between the baseline exposure $X_{0}$ and the baseline outcome $Y_{0}$. Where the association between $X_{0}$ and $Y_{0}$ is trivial, the association between $X_{0}$ and ΔY will converge on the association between $X_{0}$ and $Y_{1}$ because, when $X_{0}⊥\;Y_{0}$, then $\beta_{1}=\;\left\{E\left[Y_{1}|do\left(X_{0}=x_{0}\right)\right]-E\left[Y_{1}|do\left(X_{0}={\overset{´}{x}}_{0}\right)\right]-E\left[Y_{0}|do\left(X_{0}=x_{0}\right)\right]+\;E\left[Y_{0}|do\left(X_{0}={\overset{´}{x}}_{0}\right)\right]\right\}=\left\{E\left[Y_{1}|do\left(X_{0}=x_{0}\right)\right]-E\left[Y_{1}|do\left(X_{0}={\overset{´}{x}}_{0}\right)\right]-E\left[Y_{0}\right]+\;E\left[Y_{0}\right]\right\}=\left\{E\left[Y_{1}|do\left(X_{0}=x_{0}\right),Y_{0}=y_{0}\right]-\;E\left[Y_{1}|do\left(X_{0}={\overset{´}{x}}_{0}\right),Y_{0}=y_{0}\right]\right\}=\;\alpha_{1}$. This would be expected in large, well-conducted randomized experimental studies, in which change-score analyses may be used without invoking inferential bias (see Figure 2, panel A).

However, as the association between $X_{0}$ and $Y_{0}$ strengthens—as in non-randomized, non-experimental (i.e. observational) settings—the association between $X_{0}$ and ΔY will be increasingly dominated by the component ‘$-Y_{0}$’ and the spurious $E\left[Y_{0}|do\left(X_{0}={\overset{´}{x}}_{0}\right)\right]-E\left[Y_{0}|do\left(X_{0}=x_{0}\right)\right]$ components of the estimand, thereby diverging from the association between $X_{0}$ and $Y_{1}$. Whilst ${\hat{\beta }}_{1}$ provides a *statistically* unbiased estimate of $\beta_{1}$, it may nevertheless invoke serious inferential bias if misinterpreted as estimating $\alpha_{1}$, since the divergence between $\alpha_{1}$ and $\beta_{1}$ can be substantial and even sign-discordant. For example, if $X_{0}$ and $Y_{0}$ share a strong *positive* correlation, the negative transformation of $Y_{0}$ in the change score may dominate a smaller *positive* correlation between $X_{0}$ and $Y_{1}$, resulting in an overall *negative* association between $X_{0}$ in ΔY.

### Exogenous change vs total causal effects

It may be tempting to conclude that $\alpha_{1}\;$is always the estimand of interest in analyses of change and a *follow-up adjusted for baseline analysis* will therefore always provide the best solution where an association between $X_{0}$ and $Y_{0}$ is expected. Consideration must, however, also be given to the *direction* of the causal relationship between $X_{0}$ and $Y_{0}$, and the implications for which estimand(s) delivers the most useful causal effect(s).

The randomized experimental setting is unique for ensuring that $X_{0}$ occurs at the same time or after $Y_{0}$ by design. This guarantees that all changes in Y that are caused by $X_{0}$ will be fully realized by the effect of $X_{0}$ on $Y_{1}$. In other words, the experimental setting ensures that the effect of $X_{0}$ on exogenous change in Y is equal to the total causal effect of $X_{0}$ on $Y_{1}$ because $\left\{E\left[Y_{1}|do\left(X_{0}=x_{0}\right),Y_{0}=y_{0}\right]-E\left[Y_{1}|do\left(X_{0}={\overset{´}{x}}_{0}\right),Y_{0}=y_{0}\right]\right\}=\left\{E\left[Y_{1}|do\left(X_{0}=x_{0}\right)\right]-E\left[Y_{1}|do\left(X_{0}={\overset{´}{x}}_{0}\right)\right]\right\}\;$when $X_{0}⊥\;Y_{0}$. However, this cannot be generalized to all observational settings.

In some non-randomized contexts, such as where the baseline exposure is fast-acting and/or weakly autocorrelated over time, it may be obvious that $X_{0}$ occurs after $Y_{0}$, and that the dominant direction of causality therefore flows from $Y_{0}$ to $X_{0}$ (see Figure 2, panel B). In this setting, the effect of $X_{0}$ on exogenous change in Y again corresponds to the total causal effect of $X_{0}$ on $Y_{1}$, and a *follow-up adjusted for baseline analysis*—to estimate $\left\{E\left[Y_{1}|do\left(X_{0}=x_{0}\right),Y_{0}=y_{0}\right]-E\left[Y_{1}|do\left(X_{0}={\overset{´}{x}}_{0}\right),Y_{0}=y_{0}\right]\right\}$—is appropriate (and necessary), since $Y_{0}$ is a classical confounder for the effect of $X_{0}$ on $Y_{1}$.

However, in many other contexts, it is plausible that the baseline exposure causes both the baseline values of the outcome and the follow-up values of the outcome, due to delayed or prolonged causal effects. In such circumstances, the dominant direction of causality flows from $X_{0}$ to $Y_{0}$, and $X_{0}$ causes Y due to its effects on *both* $Y_{0}$ and $Y_{1}$(see Figure 2, panel C). In this context, the effect of $X_{0}$ on exogenous change in Y—i.e. $\alpha_{1}=\;\left\{E\left[Y_{1}|do\left(X_{0}=x_{0}\right),Y_{0}=y_{0}\right]-\;E\left[Y_{1}|do\left(X_{0}={\overset{´}{x}}_{0}\right),\;Y_{0}=y_{0}\right]\right\}$—is arguably less meaningful, since it only captures the *direct effect* of $X_{0}$ on $Y_{1}$. If this effect is sought, then a *follow-up adjusted for baseline analysis* may be appropriate—though such a strategy would involve conditioning on the mediator $Y_{0}$, which introduces additional methodological challenges.^11^^,^^12^ However, if it is the total causal effect that is sought, then a **follow-up unadjusted for baseline analysis** should be conducted to estimate $\gamma_{1}=\;\left\{E\left[Y_{1}|do\left(X_{0}=x_{0}\right)\right]-E\left[Y_{1}|do\left(X_{0}={\overset{´}{x}}_{0}\right)\right]\right\}$. This would involve constructing, e.g., a regression model of the form $\hat{Y_{1}}={\hat{\gamma }}_{0}+{\hat{\gamma }}_{1}X_{0}$, where ${\hat{\gamma }}_{1}$ represents the estimate for the estimand ($\gamma_{1}$) of interest.

The choice of whether to adjust for the baseline outcome (i.e. $Y_{0}$) is therefore *context-dependent*, as it depends upon the hypothesized causal relationship between the baseline exposure and outcome, in particular whether $Y_{0}$ is a confounder and which causal effect ($\alpha_{1}\;$or $\gamma_{1}$) is of most interest.

### Illustrative example

To illustrate the inferential bias that may be introduced from naïve analyses of change scores, we consider the causal effects of waist circumference (WC) on (log-transformed) serum insulin concentration (IC) at two times points in US adults aged 18–49 years from 2009 to 2014.^13^

## Methods

**Figure 3.**
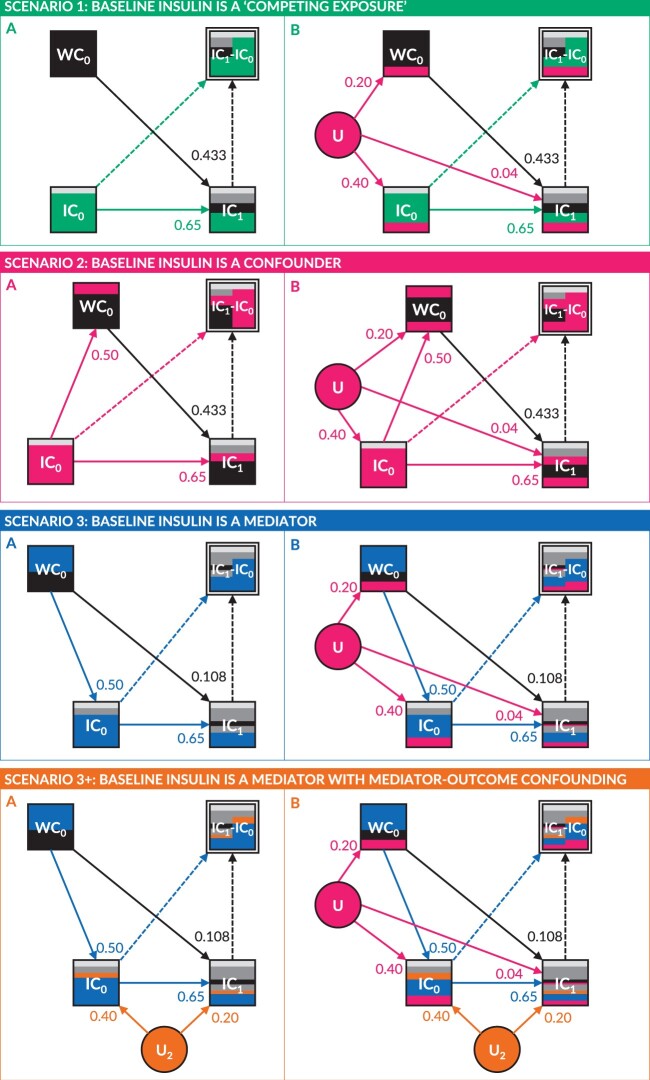
Directed acyclic graphs (DAGs) of the eight simulated scenarios. For ease of illustration, the exogenous change variable (C_1_) is not explicitly depicted, but is implicitly encapsulated within log insulin concentration at follow-up (${IC}_{1}$). ${IC}_{1}$, waist circumference at baseline (${WC}_{0}$), log insulin concentration at baseline (${IC}_{0}$), one or more unobserved confounding variables (U) and one or more unobserved mediator–outcome confounding variables ($U_{2}$) were simulated with the specified path coefficients; for more details, see the Supplementary Materials, available as Supplementary data at *IJE* online. Composite change-score variables (${IC}_{1}-{IC}_{0}$) were derived and are therefore depicted as a double-outlined nodes with dashed incoming arcs, to indicate that these were not simulated. The standardized total causal effect of ${WC}_{0}$ on ${IC}_{1}$ was fixed at 0.433, as this corresponded to a regression coefficient of 0.200 Log[mmol/L]/dm. When mediated through ${IC}_{0}$, the standardized direct effect of ${WC}_{0}$ on ${IC}_{1}$ was fixed at 0.108, as this corresponded to a regression coefficient of 0.05 Log[mmol/L]/dm.

Data were simulated to match eight simplified causal scenarios (see Figure 3):

*IC* at baseline (*IC*_0_) is neither caused by; nor the cause of; *WC* at baseline (*WC*_0_); making it a ‘competing exposure’ for the effect of *WC*_0_ on follow – up *IC* (*IC*_1_).
  - No unmeasured confounding.
  - Unmeasured variable (*U*) affecting all three source variables.
*IC* at baseline (*IC*_0_) affects *WC* at baseline (*WC*_0_); making it a confounder for the effect of *WC*_0_ on follow – up *IC* (*IC*_1_).
  - No unmeasured confounding.
  - Unmeasured variable (*U*) affecting all three source variables.
*IC* at baseline (*IC*_0_) is affected by *WC* at baseline (*WC*_0_); making it a mediator for the effect of *WC*_0_ on follow – up *IC* (*IC*_1_).
  A. No unmeasured confounding.
  B+. Unmeasured variable ($U_{2}$) affecting ${IC}_{0}$ and ${IC}_{1}$ (i.e. ‘mediator–outcome confounding’^12^).
  B. Unmeasured variable (U) affecting all three source variables.
  B+. Unmeasured variable (U) affecting all three source variables, and unmeasured variable ($U_{2}$) affecting ${IC}_{0}$ and ${IC}_{1}$ (i.e. ‘mediator–outcome confounding’).

Parameter values were informed by data from the US National Health and Nutrition Examination Survey (NHANES), for the years 2009–2014.^13^ The total causal effect of ${WC}_{0}$ on ${IC}_{1}$ was fixed at 0.200 Log[mmol/L]/dm; when mediated through ${IC}_{0}$, this was partitioned into an indirect causal effect of 0.150 Log[mmol/L]/dm and a direct causal effect of 0.050 Log[mmol/L]/dm. Full details of the simulation are provided in the Supplementary Material, available as Supplementary data at *IJE* online.

For each scenario, we then conducted three analyses using the resulting data:

1. A change-score analysis: $\hat{\Delta IC}={\hat{\beta }}_{0}+{\hat{\beta }}_{1}{WC}_{0}$.
2. A follow-up adjusted for baseline analysis: $\hat{{IC}_{1}}={\hat{\alpha }}_{0}+{\hat{\alpha }}_{1}{WC}_{0}+{\hat{\alpha }}_{2}{IC}_{0}$.
3. A follow-up unadjusted for baseline analysis: $\hat{{IC}_{1}}={\hat{\gamma }}_{0}+{\hat{\gamma }}_{1}{WC}_{0}$.

We consider the resulting regression coefficients for ${WC}_{0}$ (i.e. ${\hat{\beta }}_{1}$, ${\hat{\alpha }}_{1}$ or ${\hat{\gamma }}_{1}$) and how they relate to the causal effects of interest. To demonstrate the impact of unmeasured confounding by U and $U_{2}$ in Scenarios 1B, 2B, 3A+, 3B and 3B+, we do not explicitly adjust for these variables. Coefficient units (i.e. Log[mmol/L]/dm) are omitted to aid readability.

## Results

The resulting regression coefficients of ${WC}_{0}$ for each of the three methods of analysis for each of the three scenarios are summarized in Table 1.

**Table 1:** Regression coefficients (and 95% simulation limits) returned from three analytical approaches to estimating the ‘effect’ of waist circumference on ‘change’ in a (log) insulin concentration for the eight causal scenarios shown in Figure 3

Analysis approach	Regression coefficient for ${WC}_{0}$ (Log[mmol/L]/dm) (95% simulation limits)							
	${IC}_{0}$ is competing exposure		${IC}_{0}$ is confounder		${IC}_{0}$ is mediator			
	Scenario 1A	Scenario 1B	Scenario 2A	Scenario 2B	Scenario 3A	Scenario 3B	Scenario 3A+	Scenario 3B+
Change score ($\hat{\Delta IC}={\hat{\beta }}_{0}+{\hat{\beta }}_{1}{WC}_{0}$)	0.200 (0.180, 0.221)	0.191 (0.172, 0.210)	0.119 (0.106, 0.132)	0.114 (0.104, 0.123)	−0.031 (−0.053, −0.009)	−0.040 (−0.061, −0.019)	−0.031 (−0.050 −0.012)	−0.040 (−0.058, −0.023)
Follow-up adjusted for baseline ($\hat{{IC}_{1}}={\hat{\alpha }}_{0}+{\hat{\alpha }}_{1}{WC}_{0}+{\hat{\alpha }}_{2}{IC}_{0}$)	0.200 (0.182, 0.218)	0.203 (0.187, 0.220)	0.200 (0.189, 0.211)	0.205 (0.199, 0.211)	0.050 (0.026, 0.073)	0.047 (0.024, 0.071)	0.025 (0.005, 0.046)	0.015 (−0.005, 0.036)
Follow-up unadjusted for baseline ($\hat{{IC}_{1}}={\hat{\gamma }}_{0}+{\hat{\gamma }}_{1}{WC}_{0}$)	0.200 (0.174, 0.226)	0.228 (0.203, 0.253)	0.351 (0.332, 0.369)	0.382 (0.366, 0.398)	0.200 (0.175, 0.226)	0.228 (0.203, 0.253)	0.200 (0.174, 0.226)	0.228 (0.203, 0.253)

${\mathrm{WC}}_{0}$ , waist circumference at baseline; ${\mathrm{IC}}_{0}$, (Log) insulin concentration at baseline; ${\mathrm{IC}}_{1}$, (Log) insulin concentration at follow-up; ΔIC, (Log) insulin concentration change score (${\mathrm{IC}}_{1}-{\mathrm{IC}}_{0}$). The total causal effect of ${\mathrm{WC}}_{0}$ on ${\mathrm{IC}}_{1}$ was simulated to be 0.200 Log[mmol/L]/dm. When mediated through ${\mathrm{IC}}_{0}$, this was partitioned into an indirect causal effect of 0.150 Log[mmol/L]/dm and a direct causal effect of 0.050 Log[mmol/L]/dm. Deviations from these values reflect statistical or inferential bias.

***(i) Scenario 1: Baseline insulin is a ‘competing exposure’ (*** *i.e.* ***is neither caused by, nor the cause of, baseline waist circumference)***

In Scenario 1:

-
$\;\alpha_{1}\;=\;\beta_{1}=\;\gamma_{1}\;=\;0.200\;=\;\mathrm{the}\;\mathrm{total}\;\mathrm{causal}\;\mathrm{effect}\;\mathrm{of}\;{WC}_{0}\;\mathrm{on}\;{IC}_{1}\;=\;\mathrm{the}\;\mathrm{effect}\;\mathrm{of}\;{WC}_{0}\;\mathrm{on}\;\mathrm{exogenous}\;\mathrm{change}\;\mathrm{in}\;IC$

Scenario 1A is analogous to a large, well-conducted randomized experimental study. The association between ${WC}_{0}$ and ΔIC thus consists entirely of the causal effect of ${WC}_{0}$ on ${IC}_{1}$. Since there is no confounding or mediation by ${IC}_{0}$, all methods of analysis provide an unbiased estimate of the causal effect of ${WC}_{0}$ on exogenous change in IC (${\hat{\beta }}_{1}={\hat{\alpha }}_{1}={\hat{\gamma }}_{1}=0.200$).

In Scenario 1B, the association between ${WC}_{0}$ and ΔIC again consists of the causal effect of ${WC}_{0}$ on ${IC}_{1}$ but this is now confounded by U. All three methods of analysis provide a biased estimate of the causal effect of ${WC}_{0}$ (${\hat{\beta }}_{1}=0.191$, ${\hat{\alpha }}_{1}=0.203$, ${\hat{\gamma }}_{1}=0.228$). However, it is worth noting that the *follow-up adjusted for baseline* estimate (i.e. ${\hat{\alpha }}_{1}$) is less biased than the *follow-up unadjusted for baseline estimate* (i.e. ${\hat{\gamma }}_{1}$), since adjustment for ${IC}_{0}$ closes one of the two confounding paths between ${WC}_{0}$ and ${IC}_{1}$.

***(ii) Scenario 2: Baseline insulin is a confounder***

In Scenario 2:

-
$\alpha_{1}\;=\;0.200\;=\;\mathrm{the}\;\mathrm{total}\;\mathrm{causal}\;\mathrm{effect}\;\mathrm{of}\;{WC}_{0}\;\mathrm{on}\;{IC}_{1}\;=\;\mathrm{the}\;\mathrm{effect}\;\mathrm{of}\;{WC}_{0}\;\mathrm{on}\;\mathrm{exogenous}\;\mathrm{change}\;\mathrm{in}\;IC$

In Scenario 2A, the association between ${WC}_{0}$ and ΔIC consists of the causal effect of ${WC}_{0}$ on ${IC}_{1}$ and confounding by ${IC}_{0}$. Both the *change-score analysis* and *follow-up unadjusted for baseline analysis* provide biased estimates of the causal effect of ${WC}_{0}$ on exogenous change in IC (${\hat{\beta }}_{1}=0.119$ and ${\hat{\gamma }}_{1}=0.351$, respectively). The *follow-up adjusted for baseline analysis* recovers the correct total causal effect (${\hat{\alpha }}_{1}=0.200$) because conditioning on ${IC}_{0}$ closes the confounding path through ${IC}_{0}$.

In Scenario 2B, the association between ${WC}_{0}$ and ΔIC consists of the causal effect of ${WC}_{0}$ on ${IC}_{1}$ and confounding from both ${IC}_{0}$ and U. All methods of analysis provide a biased estimate of the causal effect of ${WC}_{0}$ (${\hat{\beta }}_{1}=0.114$, ${\hat{\alpha }}_{1}=0.205$, ${\hat{\gamma }}_{1}=0.382$), though the *follow-up adjusted for baseline analysis* remains the least biased.

***(iii) Scenario 3: Baseline insulin is a mediator***

In Scenario 3:

-
$\;\alpha_{1}\;=\;0.050\;=\;\mathrm{the}\;\mathrm{direct}\;\mathrm{causal}\;\mathrm{effect}\;\mathrm{of}\;{WC}_{0}\;\mathrm{on}\;{IC}_{1}\;=\;\mathrm{the}\;\mathrm{effect}\;\mathrm{of}\;{WC}_{0}\;\mathrm{on}\;\mathrm{exogenous}\;\mathrm{change}\;\mathrm{in}\;IC$

-
$\;\gamma_{1}\;=\;0.200\;=\;\mathrm{the}\;\mathrm{total}\;\mathrm{causal}\;\mathrm{effect}\;\mathrm{of}\;{WC}_{0}\;\mathrm{on}\;{IC}_{1}$

In Scenario 3A, the association between ${WC}_{0}$ and ΔIC consists of both the direct causal effect of ${WC}_{0}$ on ${IC}_{1}$  *and* the indirect causal effect that is mediated through ${IC}_{0}$. The *change-score analysis* (${\hat{\beta }}_{1}=-0.031$) provides a biased estimate of opposite sign to both the direct causal effect ($\alpha_{1}$) of ${WC}_{0}$ on ${IC}_{1}$ (equivalent to the effect of ${WC}_{0}$ on exogenous change in IC) and the total causal effect ($\gamma_{1}$) of ${WC}_{0}$ on ${IC}_{1}$. The *follow-up adjusted for baseline analysis* provides an unbiased estimate of the direct causal effect of ${WC}_{0}$ on ${IC}_{1}$ (${\hat{\alpha }}_{1}=0.050$), though the estimate is biased (${\hat{\alpha }}_{1}=0.025$) in the presence of mediator–outcome confounding (Scenario 3A+), since conditioning on ${IC}_{0}$ opens a confounding path through $U_{2}$.^12^ The *follow-up unadjusted for baseline analysis* provides an unbiased estimate of the total causal effect of ${WC}_{0}$ on ${IC}_{1}$ (${\hat{\gamma }}_{1}=0.200$), which remains robust in the presence of mediator–outcome confounding (Scenario 3A+).

In Scenario 3B, as previously, the association between ${WC}_{0}$and ΔIC again consists of the direct causal effect of ${WC}_{0}$on ${IC}_{1}$ and the indirect causal effect mediated through ${IC}_{0}$, but this is now confounded by U. The *change-score analysis* remains biased (${\hat{\beta }}_{1}=-0.031$) and with the opposite sign to both the direct and total causal effects. Both the *follow-up adjusted for baseline analysis* and *follow-up unadjusted for baseline analysis* provide biased estimates of the direct causal effect (${\hat{\alpha }}_{1}=0.047$) and total causal effect (${\hat{\gamma }}_{1}=0.228$) of ${WC}_{0}$, respectively. The bias of the *follow-up adjusted for baseline analysis* is exacerbated (${\hat{\gamma }}_{1}=0.015$) in the presence of mediator–outcome confounding (Scenario 3B+) due to conditioning on the collider ${IC}_{0}$.

## Discussion

Our study explains why analyses of change scores do not estimate causal effects in observational data. To demonstrate, we explored the ostensibly simple context of analysis of change in an outcome (insulin concentration) with respect to a baseline exposure (waist circumference) for eight different causal scenarios. Misleading coefficients, sometimes of opposite sign to the true effects of interest, were observed in every scenario except where the baseline outcome was a ‘competing exposure’, i.e. was neither caused by, nor the cause of, the baseline exposure. Although such independence is plausible, and is indeed actively sought in randomized experimental studies, it is extremely unlikely when the exposure is not assigned randomly. Many analyses of change scores in observational studies are therefore likely to suffer inferential bias, the size of which will vary with the strength and nature of the association between the baseline exposure and baseline outcome.

### Recommendations

Analyses of outcome-change scores to estimate causal effects in observational data should be avoided, including ‘percentage’-change scores, where the change between baseline and follow-up is expressed as a percentage of the baseline value. If the follow-up outcome is not normally distributed, appropriate transformations and/or non-parametric methods should be preferred to calculating and analysing change scores.^14^

Ideally, all causal effect(s) of interest should be formally identified using DAGs and estimated accordingly. We believe the total causal effect of the baseline exposure (i.e. $X_{0}$) on the follow-up outcome (i.e. $Y_{1}$) will generally offer the greatest interest and utility, as it provides the simplest summary of how changing the exposure would be expected to change future values of the outcome. Where the baseline outcome (i.e. $Y_{0}$) is a ‘competing exposure’ or confounder for the effect of the exposure on the follow-up outcome, the total causal effect of the exposure on the follow-up outcome is the same as its effect on exogenous change in the outcome. Where the baseline outcome is a mediator for the effect of the exposure on the follow-up outcome, the direct causal effect of the exposure on the follow-up outcome captures its effect on exogenous change in the outcome. If the direct causal effect is sought, estimating this will need to account for potential mediator–outcome confounding.^11^^,^^12^

### Caveats

Not all uses of outcome-change scores will necessarily produce incorrect or misleading estimates. Change scores may provide a robust summary of the average change in a variable between two time points for a group or individual; problems only arise when statistical comparisons are made either between groups or individuals, or in relation to one or more other variables. Change scores may therefore still be qualitatively useful for tracking the progress of individuals, provided it is recognized that the magnitude of any expected change is functionally determined by the baseline value.

Where the exposure is unrelated to the outcome at baseline (such as in randomized experimental studies), analyses of change scores provide unbiased estimates. However, even under these circumstances, analyses of change scores are less efficient than follow-up adjusted for baseline analyses (e.g. ANCOVA), unless the change-score analysis is also adjusted for the baseline outcome.^15^ In fact, analyses of change scores that adjust for the baseline outcome (i.e. *change score adjusted for baseline analyses*) can provide unbiased estimates even in non-randomized data, because they are mathematically identical to follow-up adjusted for baseline analyses. This is because adjusting for $Y_{0}$ eliminates the contribution of the '${-Y}_{0}$' component in the outcome, i.e. $\left[\left(Y_{1}-Y_{0}\right)|Y_{0}]={[Y}_{1}|Y_{0}\right]$.^17^^,^^18^ However, extra care must be taken to avoid interpreting the coefficient for the baseline outcome as a model covariate, as this will primarily reflect the tautological association with the change score.

In some situations, the coefficient of a change-score analysis (${\hat{\beta }}_{1}$) may coincide with the desired estimand ($\alpha_{1}$) if the spurious elements of the change-score estimand happen to equal all other unobserved confounding^19^ or else provide less biased ‘estimates’ than the appropriate estimator. Unfortunately, since it is impossible to know when such situations occur, it is inconceivable that this may ever offer practical utility.

Even when adopting a robust analytical strategy, analyses of change with only two measurements will almost always produce inaccurate effect estimates due to random variation (whether error or otherwise) in the baseline and/or follow-up measures. A diluted estimate can be expected because it is not possible to distinguish between the (desired) effect on exogenous change from the association with the random determinants of change (which will average at zero). Some information about the random variation can, however, be gained from the baseline outcome and this explains why adjusting for the baseline outcome (e.g. using ANCOVA) offers improved precision over unconditional analyses of the follow-up outcome in randomized experimental data. In observational data, this benefit is secondary to considering the causal relationship between $X_{0}$ and $Y_{0}$. When $Y_{0}\;$is a confounder for the effect of $X_{0}$ on $Y_{1}$ (and hence ‘change’ in Y), reducing this confounding through conditioning is theoretically appropriate and necessary. However, some residual confounding will remain because it is not possible to distinguish between the ‘stable’ or structural features of $Y_{0}$ (that may cause $Y_{1}$) and the random features (that cannot cause $Y_{1}$). Change scores cannot offer a solution to these consequences of limited measurement, since they contain no additional information than their parent variables $Y_{0}$ and $Y_{1}$.

Additional measurements are necessary to reduce the issues with random variation. Latent variable methods provide an elegant means to summarize the pattern of growth over multiple time points, although care must be taken to avoid other inferential biases due to regression-to-the-mean.^20^ When used appropriately, latent growth-curve models avoid the same problems as change-score analyses because they are centred across all datapoints, ensuring the intercept and slope do not share the same spurious negative correlation as in analyses of change scores. This is conceptually similar to Oldham's suggestion that change between baseline and follow-up be compared against the mean of the two values [i.e. $\left(Y_{0}+Y_{1}\right)/2$]^2^—the same approach as recommended by Bland and Altman for calculating limits of agreement.^20^ Summary features, such as ‘slope’, nevertheless still possess some conceptual challenges, due to the conflation of causal information from multiple time points.^21^

### Ontology of change

Whether analyses of change are meaningful or misleading is ultimately a matter of ontology, since the problems that arise are inferential, not statistical. We conceptualize three reasons for a variable changing value over time. The first, ‘determined change’, is not really change, but the realization of a past event at a later point in time. This is analogous to the inevitable future consequences of a present event within space-time.^22^ The second, ‘random change’, represents all the random reasons for a variable changing value beyond what has been determined. Strictly, this consists of all uncertainty arising from the quantum, although, pragmatically, it will also include all apparently random behaviour arising from intractable complexity.^23^ Finally, ‘exogenous change’ represents all non-random reasons for a variable changing value beyond what has been determined. This is analogous to the influence of all events in the ‘absolute elsewhere’ within space-time.^22^ Of these three reasons for a variable changing value, exogenous change offers the only route to external influence, making it the principal interest of causal enquiry.

### Study limitations

Our simulations were deliberately simplified and made several distributional assumptions that may not be entirely realistic. Multiple variables are likely to confound the true causal effect of waist circumference on insulin concentration. Rather than simulating these individually, we simulated a single summary confounder U for illustrative purposes. The focus of this paper was not, however, on one specific context; rather, we sought to demonstrate the potential problems with analysing and interpreting change scores in observational studies and the utility of DAGs for exploring and identifying such issues. No inferences should be drawn from our simulations about the assumed causal effect of waist circumference on insulin concentration, which may not exist. We did not consider the additional complications that would result from non-linear relationships, where change scores and linear conditioning for the baseline outcome (e.g. using ANCOVA) would introduce further bias. Where confounding is present and conditioning is required, appropriate parameterization should be sought to reduce residual confounding.

### Comparison with Lord (1967) and Glymour *et al.* (2005)

Scenario 3A, in which the baseline outcome mediates the effect of the exposure on the follow-up outcome, represents the same situation that originally puzzled Lord in 1967.^5^ Lord's confusion arose because the *change-score analysis* and *follow-up adjusted for baseline analysis* produced very different results, neither of which seemed to resolve the ‘pre-existing’ differences in weight at baseline. Using a causal perspective, we can recognize that this ‘paradox’ occurred for two distinct reasons: (i) *follow-up adjusted for baseline analyses* do not provide *total causal effect*s because the baseline outcome is a mediator and (ii) *change-score analyses* do not provide meaningful causal-effect estimates in observational data. Although these points have been individually recognized elsewhere,^7^^,^^24^ they have not yet been explicitly recognized jointly.

Our conclusion that change scores do not estimate causal effects in non-randomized contexts, including any effect on ‘exogenous’ change, may explain the divergence between our conclusions and those of Glymour *et al.*^6^ Glymour *et al.*'s study compares two change-score analyses: one *with* and one *without* adjustment for a mediating baseline outcome. However, as discussed above, *change score adjusted for baseline analyses* are equivalent to *follow-up adjusted for baseline analyses*,^17^^,^^18^ meaning that the scenario in Glymour *et al.* mirrored Lord's paradox and gave similarly divergent results. Glymour *et al.* attributed this divergence to the introduction of measurement error when adjusting for the baseline outcome and concluded that ‘*change-score analyses without baseline adjustment provide unbiased causal effect estimates*’.^6^ We suspect that the difference instead reflects the differing estimands, with only the *change score adjusted for baseline analyses* returning a potentially meaningful estimand—the *direct causal effect*.

## Conclusion

Judgements regarding clinical significance and the funding and delivery of treatment are dependent on obtaining meaningful causal-effect estimates, and analyses of outcome-change scores in non-randomized data do not provide this. Moreover, such analyses may even suggest an ‘effect’ that is of the opposite sign to the total causal effect. Observational studies that have analysed outcome-change scores should therefore be viewed with caution and their recommendations revisited.

## Supplementary data

Supplementary data are available at *IJE* online.

## Ethics approval

Ethics approval was not required for this research, as it did not involve human subjects.

## Funding

This study received no specific funding. P.W.G.T., K.F.A. and M.S.G. are supported by the Alan Turing Institute [grant number EP/N510129/1].

## Supplementary Material

dyab050_Supplementary_Data

## Data Availability

The simulation code is available on Github at https://github.com/pwgtennant/change-score.
