# Supplementary material for: Analyses of ‘change scores’ do not estimate causal effects in observational data
Source: Int J Epidemiol. 2021 Jun 7;51(5):1604–15. doi: 10.1093/ije/dyab050 (PMC9557845; doi:10.1093/ije/dyab050)
Supplement: dyab050_Supplementary_Data [file dyab050_supplementary_data.docx]

SUPPLEMENTARY MATERIAL

**Analyses of 'change scores' do not estimate causal effects in observational data**

# CONTENTS

[SUPPLEMENTARY METHODS 2](#_Toc62813048)

[Data simulation 2](#_Toc62813049)

[SUPPLEMENTARY TABLES 3](#_Toc62813050)

[Supplementary Table S1 3](#_Toc62813051)

[SUPPLEMENTARY REFERENCES 4](#_Toc62813052)

#

# SUPPLEMENTARY METHODS

## Data simulation

Data were simulated to match the eight causal scenarios depicted in Figure 3, with parameter values and path coefficients informed by data from the US National Health and Nutrition Examination Survey (NHANES), for the years 2009-2014 (Table A.1).^25^

Since insulin concentration ($IC$) appears log-normally distributed,^26^ we simulated and analysed $IC$ in log-transformed form. For each scenario, multivariate normal data with values for baseline $IC$ (${IC}_{0}$), follow-up $IC$ (${IC}_{1}$), and baseline $WC$ (${WC}_{0}$) were simulated in a sample of 1000 participants using ‘dagitty’ v.0.2.2 in R 3.4.0.^27,28^ The simulated mean (SD) for ${IC}_{0}$ was 4.00 Log[mmol/L] (0.74) and 4.20 Log[mmol/L] (0.74) for ${IC}_{1}$, representing a notional 5% increase from baseline to follow-up. The simulated mean (SD) for ${WC}_{0}$ was 9.5dm (1.6). Path coefficients between these variables were selected to reflect the observed cross-sectional correlation of $r\approx0.6$ (Table A.1).^25^ The path coefficient between ${IC}_{0}$ and ${IC}_{1}$ was simulated as 0.65, to represent strong but imperfect determination over time. In some scenarios, one or more unobserved confounding factors were represented by $U$, which was simulated to introduce a confounded correlation of $r\approx0.08$ between ${WC}_{0}$ and both ${IC}_{0}$ and ${IC}_{1}$. The total causal effect of ${WC}_{0}$ on ${IC}_{1}$ was fixed at 0.2 Log[mmol/L]/dm, equivalent to a direct path coefficient of 0.433. When mediated through ${IC}_{0}$, this was partitioned into an indirect causal effect of 0.15 Log[mmol/L]/dm and a direct causal effect of 0.05 Log[mmol/L]/dm. For illustrative purposes, we also simulated mediator-outcome confounding ($U_{2}$) averaging $r\approx0.08$ between ${IC}_{0}$ and ${IC}_{1}$. For each scenario, simulations were repeated 10,000 times^29^ and the regression coefficients for ${WC}_{0}$ stored. Median values are reported with their 95% simulation limits (2.5 and 97.5 centile estimates from the 10,000 samples).

# SUPPLEMENTARY TABLES

## Supplementary Table S1

Observed and simulated means, standard deviations (SD), and Pearson correlation coefficients for waist circumference and insulin concentration. Observed values are from the US National Health and Nutrition Examination Survey (NHANES), for the years 2009-2014.

|  | ┌─────────────────── Mean (SD) ───────────────────┐ | | | |
| --- | --- | --- | --- | --- |
|  | ┌─────── Reported by NHANES ──────┐ | | | Simulated |
|  | 2009-2010 | 2011-2012 | 2013-2014 |  |
| Waist circumference (dm) | 9.50 (1.58) | 9.42 (1.61) | 9.52 (1.65) | 9.50 (1.60) |
| Insulin concentration (Log[mmol/L]) | 4.20 (0.70) | 4.08 (0.74) | 3.98 (0.77) | Baseline: 4.00 (0.74) Follow-up: 4.20 (0.74) |
| Pearson correlation (ρ) | 0.58^a^ | 0.58^a^ | 0.60^a^ | 0.50 - 0.60 |

^a^Between waist circumference (WC) and log insulin concentration (IC).

# SUPPLEMENTARY REFERENCES

1. National Center for Health Statistics. National Health and Nutrition Examination Survey, 2009–2014 Data Files. Hyattsville (USA): Centers for Disease Control and Prevention; 2016. <https://www.cdc.gov/nchs/nhanes/>.
2. Statistics NCfH. National Health and Nutrition Examination Survey: 2013-2014 Insulin Data Documentation, Codebook, and Frequencies. Hyattsville (USA): Centers for Disease Control and Prevention; 2016: <https://wwwn.cdc.gov/Nchs/Nhanes/2013-2014/INS_H.htm>.
3. R Core Team. R: A language and environment for statistical computing. Vienna, Austria: R Foundation for Statistical Computing; 2014: <http://www.R-project.org/>.
4. Textor J, van der Zander B, Gilthorpe MS, Liskiewicz M, Ellison GT. Robust causal inference using directed acyclic graphs: the R package 'dagitty'. *Int J Epidemiol.* 2016;45(6):1887-1894.
5. Burton A, Altman DG, Royston P, Holder RL. The design of simulation studies in medical statistics. Stat Med. 2006;25(24):4279-4292.
